# Country-specific modifiable dementia risk factors across the Western Pacific Region determined by population attributable fraction

**DOI:** 10.1016/j.lanwpc.2026.101857

**Published:** 2026-04-17

**Authors:** Claire V. Burley, Hamid R. Sohrabi, Maha Alshahrani, Jennifer Dunne, Sharon L. Naismith, Kaarin J. Anstey, Tanya Buchanan, Mario Siervo, Blossom C.M. Stephan

**Affiliations:** a Dementia Centre of Excellence, enAble Institute, Curtin University, Perth, Western Australia, Australia; b School of Health Sciences, University of New South Wales, Kensington, New South Wales, Australia; c Centre for Healthy Ageing, Health Futures Institute, Murdoch University, Perth, Western Australia, Australia; d School of Psychology, Murdoch University, Perth, Western Australia, Australia; e Brain and Mind Centre, The University of Sydney, Sydney, New South Wales, Australia; f School of Psychology, University of New South Wales, Kensington, New South Wales, Australia; g Neuroscience Research Australia, 139 Barker St, Randwick, 2031, Australia; h University of New South Wales, Ageing Futures Institute, Kensington, New South Wales, Australia; i Dementia Australia, North Ryde, New South Wales, Australia; j School of Population Health, Curtin University, Perth, Australia

**Keywords:** Dementia, Risk factor, Health equity

## Abstract

**Background:**

One-third of global dementia cases occur in the Western Pacific Region (WPR), a region of marked socioeconomic and cultural diversity. Understanding how modifiable risk factors contribute to dementia burden across countries is essential for designing context-specific prevention strategies. This study quantified country-level variability in dementia cases attributable to modifiable risk factors across the WPR.

**Methods:**

Weighted population attributable fractions (PAFs) for nine modifiable factors: low education, obesity, physical inactivity, hypertension, diabetes, smoking, hearing loss, depression, and alcohol misuse were calculated for 32 countries. For 13 countries with full datasets, combined weighted PAFs were also generated for seven of the nine factors (excluding hearing loss and alcohol misuse). PAF values were compared across high-, upper-middle-, and lower-middle-income countries to explore socioeconomic patterns.

**Findings:**

Weighted PAFs showed substantial cross-country variability. The largest variation occurred for low education (0.0–7.3%; n = 16; vs global 4.5%) and obesity (0.2–5.9%; n = 28; vs global 1.4%). Hypertension (0.9–2.2%; n = 27; vs global 2.2%) and alcohol misuse (0.0–0.5%; n = 24; vs global 1.0%) showed the least variation. Combined weighted PAFs ranged from 20.1% (Singapore) to 34.7% (Papua New Guinea). Income-related patterns emerged: low education contributed most to dementia burden in lower-middle-income countries, while diabetes and depression contributed more in upper-middle- and high-income countries.

**Interpretation:**

Eliminating seven modifiable risk factors could prevent 20–35% of dementia cases across the WPR. Region-wide gains may be achieved by focussing on diabetes and hearing loss, while effective prevention strategies require tailoring to country-specific socioeconomic conditions, health-system capacity, and education inequalities.

**Funding:**

This work was made possible through Dementia Australia's support of their Chair of Dementia (author BCMS). KJA is funded by an Australian Laureate Fellowship - Grant ID: FL190100011.

**Research in context**

**Evidence before this study**

The 2024 *Lancet* Commission on Dementia reported that 14 modifiable risk factors could prevent up to 45% of dementia cases globally, drawing primarily on evidence from high-income Western populations. Previous population attributable fraction (PAF) analyses conducted in the USA, UK, Germany, Brazil, Portugal, and Mozambique using a seven-factor model (incorporating low education, obesity, physical inactivity, hypertension, diabetes, smoking, and depression) demonstrated regional variation, with estimated prevention potential ranging from 24% to 40%. Although substantial differences in dementia risk factor profiles have been documented across Western Pacific Region (WPR) countries, no comprehensive region-wide PAF analysis has been undertaken. This gap is notable given that the WPR accounts for more than one-third of global dementia cases and is projected to experience the world's largest dementia increase by 2050.

**Added value of this study**

This study provides the first systematic, country-specific PAF analysis across the WPR. By calculating weighted PAFs for nine modifiable risk factors across 32 countries, we show pronounced heterogeneity in prevention potential, particularly for low education and obesity. Combined weighted PAFs for seven core factors ranged from 20 to 35%, comparable to estimates from the USA (28%), UK (31%), Germany (30%), Brazil (32%), Portugal (40%), and Mozambique (24%). Clear socioeconomic patterns emerged: low education, hearing loss, and hypertension contributed more substantially in lower-income settings, whereas diabetes and depression were more prominent in higher-income countries. Country-specific profiles also revealed divergent priorities, for example, smoking reduction would have the greatest impact in China, whereas increasing physical activity would yield the largest benefits in New Zealand. These findings emphasise that standardised global prevention strategies may not be optimal for the WPR.

**Implications of all the available evidence**

Dementia prevention in the WPR requires regionally adapted approaches rather than reliance on global averages. Diabetes, hearing loss, and smoking represent region-wide priorities, while educational interventions are particularly relevant for lower-income countries and mental health approaches for higher-income settings. The 24 WPR countries without a national dementia strategy should develop tailored plans informed by their risk factor profiles. Future work should evaluate culturally appropriate interventions and assess the cost-effectiveness of targeted prevention models.

## Introduction

Spanning 37 countries and home to nearly 1.9 billion people, the Western Pacific Region (WPR) is one of the most culturally, economically, and geographically diverse WHO regions.^1^^,^^2^ Like global trends, the WPR population is rapidly ageing, resulting in a rising burden of age-related disease, particularly dementia.^3^ The region currently has the third-highest number of people living with dementia worldwide (20 million out of 55 million globally), but projections indicate that by 2050 it will have the largest burden, with prevalence expected to triple to over 76 million of an estimated 139 million cases worldwide.^3^^,^^4^ This trajectory underscores the urgent need for prevention strategies tailored to the diverse risk profiles and resource settings across WPR countries. A recent systematic review of population attributable fractions (PAFs), defined as the proportion of cases that can be attributed to a given risk factor, identified 61 modifiable dementia risk factors spanning the life course including low education, hearing loss, depression, physical inactivity, diabetes, smoking, hypertension, obesity, diet, sleep and alcohol misuse.^5^ Individually, these factors account for between 5.3% (obesity) and 17.2% (low education) of global dementia burden. When risk factors are combined, the potential for prevention increases substantially. A commonly used seven-factor model incorporating low education, midlife hypertension, midlife obesity, smoking, physical inactivity, depression, and diabetes yields a pooled weighted PAF of 32.0% across 25 countries.^5^ The 2024 Lancet Commission's expanded 14-factor model incorporating early life low education, midlife hearing loss, high LDL cholesterol, depression, traumatic brain injury, physical inactivity, diabetes, smoking, hypertension and obesity, and late life social isolation, air pollution and visual loss, estimates that up to 45% of dementia cases worldwide could be prevented with comprehensive risk factor modification.^6^

However, most PAF estimates are derived from high-income Western populations of predominately white ethnicity. These estimates may not accurately represent the risk factor burden or prevention potential in non-Western regions. Within the WPR, countries differ considerably in dementia prevalence, risk factor patterns, healthcare capacity, socioeconomic conditions, and policy response.^2^ Notably, only eight of the 37 WPR countries have implemented a national dementia action plan.^3^ As such, applying global estimates uniformly may lead to misaligned priorities and suboptimal allocation of resources within the region. Building on evidence from Clarke and colleagues,^1^ which documented substantial variations in dementia risk factor profiles across WPR countries, we calculated the individual PAFs for dementia associated with nine of the 14 factors identified in the 2024 Lancet Commission^6^: low education, obesity, physical inactivity, hypertension, diabetes, smoking, hearing loss, depression, and alcohol misuse. We additionally derived combined weighted PAFs for the established seven-factor model in all countries with complete data, enabling comparison with international PAF analyses that use similar methodology.^7^, ^8^, ^9^, ^10^, ^11^ Finally, we examined differences by country income status to identify which risk factors contribute most to dementia burden in higher-income, upper-middle-income, and lower-middle-income settings, to inform targeted, resource-appropriate prevention strategies across this diverse region.

## Methods

### Data acquisition

Risk factor prevalence estimates for WPR countries were extracted from published sources.^1^ Full details of the data sources, risk factor definitions, and cut-offs, and country-level prevalence estimates are provided in Appendix Tables 1–3. Of the 14 modifiable risk factors identified by the 2024 Lancet Commission on Dementia, data were available for nine risk factors across 32 WPR countries: low education, obesity, physical inactivity, hypertension, diabetes, smoking, hearing loss, depression, and alcohol misuse. Insufficient data were available for high LDL cholesterol, traumatic brain injury, social isolation, air pollution, and untreated vision loss. Relative risk (RR) and communality values for the nine risk factors were extracted from the 2024 Lancet Commission.^6^ RR and communality values for the seven-factor model were sourced from Livingston and colleagues^6^ and Norton and colleagues^10^ to allow comparison with published estimates (Appendix Table 4).

RR and communality estimates were assumed to be constant across countries and applied uniformly to all prevalence data, consistent with prior global dementia risk modelling approaches. Communality values were used to account for non-independence between risk factors and represent the proportion of variance in a given risk factor that is shared with other included factors. They reflect correlations between risk factors and are independent of population prevalence; variation in prevalence across countries affects the magnitude of individual PAFs but not the assumed structure of risk factor overlap. In this context, communality quantifies the degree to which one risk factor may overlap with others (e.g., hypertension, diabetes, and obesity), thereby preventing double-counting of shared attributable risk when estimating combined PAFs. For combined weighted PAFs, individual PAFs were adjusted using communality weights (*w*ᵢ) such that: PAFᵢ, weighted = *w*ᵢ × PAFᵢ, where *w*ᵢ = 1 − communalityᵢ.

### Data analysis

#### Unweighted population attributable fractions

Individual unweighted PAFs were calculated using Levin's formula^12^:

$$\text{PAFi},\text{unweighted}=\frac{Pi*\left(RRi-1\right)}{Pi*\left(RRi-1\right)+1}$$

where Pi is the prevalence of risk factor i in each country and RRi is the corresponding RR value drawn from published literature as outlined above. PAFs were expressed as percentages.

For the seven-factor model, a combined unweighted PAF, assuming independent between each factor, was calculated using the multiplicative method:

$$\text{PAF}\;\text{combined},\text{unweighted}=1-\prod_{i=1}^{k}\left(1-\text{PAF}i\right)$$

This incorporated diabetes, midlife hypertension, midlife obesity, physical inactivity, depression, smoking, and low education.^10^

#### Weighted population attributable fractions

To account for intercorrelation among risk factors, weighted individual and combined PAFs were calculated by adjusting for shared variance using published communality values. Communalities were taken from the 2024 Lancet Commission on Dementia^6^ for the nine factor analysis and from Norton and colleagues^10^ for the seven-factor analysis (see Appendix Table 4). Because our prevalence data were aggregated at the country level, communalities could not be calculated directly.

Weighted individual PAFs were calculated as:

$$\text{PAFi},\text{weighted}=wi*\;\frac{Pi*\left(RRi-1\right)}{Pi*\left(RRi-1\right)+1}$$

where wi is the weight for the individual risk factor and PAFi in the individual contribution to PAF.

Combined weighted PAF values for the seven-factor model were calculated as:

$$\text{PAF}\;\text{combined},\text{weighted}=1-\prod_{i=1}^{k}\left(1-wi*\;\text{PAF}i\right)$$

Weighted PAF values (individual and for the combined seven factor model) are presented in the Results, with unweighted PAFs provided in Appendix Table 5. Combined unweighted PAFs are presented in the Supplementary Materials for transparency and comparison with previous literature but are not interpreted due to their known limitations.^5^^,^^10^

The Lancet Commission framework was used as the primary model given its contemporary relevance and comprehensive structure. The Norton seven-factor model was additionally included to enable comparison with earlier literature and to maximise inclusion of countries with sufficient data availability. Results from the Norton model are presented for contextual and comparative purposes and are not intended to supersede Lancet Commission estimates.

#### Visualisation

Heatmaps were generated to visualise individual weighted PAFs across WPR countries. A colour gradient was applied, where darker shades represent higher PAF values. Countries were displayed geographically where possible, and only those with available data for each risk factor were included. Colour scales were standardised within each risk factor to enable cross-country comparison. Countries with missing data were shown in grey or excluded from that heatmap. These visualisations enabled clear identification of geographic patterns and high- and low-burden countries for each risk factor (Appendix Fig. 1A–D).

#### Country stratification by income-level

Countries were classified by income groups using the 2024 World Bank income classification system,^13^ consistent with the source prevalence data from Clarke and colleagues.^1^ Countries were grouped into lower-middle (n = 7 countries; Cambodia, Kiribati, Lao, Micronesia, Papua New Guinea, Solomon Islands and Vanuatu), upper-middle (n = 10 countries; China, Fiji, Malaysia, Marshall Islands, Mongolia, The Philippines, Samoa, Tonga, Tuvalu and Vietnam), and high-income (n = 15 countries; American Samoa, Australia, Brunei Darussalam, Cook Islands, French Polynesia, Guam, Japan, Nauru, New Zealand, Niue, Northern Mariana Islands, Palau, Korea, Singapore and Tokelau) based on the gross national income (GNI) per capita.

Group differences in mean weighted PAFs were assessed using a one-way ANOVA. Normality of weighted PAF distributions was examined using Shapiro–Wilk tests and Q-Q plots. Most variables demonstrated significant departures from normality (overall Shapiro–Wilk p < 0.05 for most risk factors), and normality was violated within one or more income groups. Given these findings and small group sizes, non-parametric Wilcoxon rank-sum tests were used for pairwise comparisons. To account for multiple testing across pairwise comparisons within each risk factor, p-values were adjusted using the Holm–Bonferroni method, with statistical significance assessed at a familywise α of 0.05. All analyses were performed in Stata version 18.^14^

### Ethics approval

Ethics approval was not required for this study as it used only publicly available, aggregated secondary data. This was confirmed by the Human Research Ethics Committee at Curtin University.

### Role of the funding source

This work was made possible through Dementia Australia's support of their Chair of Dementia (author BCMS). KJA is funded by an Australian Laureate Fellowship - Grant ID: FL190100011. None of the funding organisations played a role in conducting the study, collecting, managing, analysing, or interpreting the data, or in the preparation, review, or approval of the manuscript.

## Results

### Sample characteristics

Data from all 32 WPR countries were included in the initial analysis calculating individual unweighted and weighted risk factor PAF values. Data completeness varied by risk factor: low education had the most missing data (17/32 countries missing), followed by depression (10/32 countries missing), alcohol misuse (8/32 countries missing) and smoking (7/32 countries missing). Full details on missing data are provided in Appendix Table 3.

**Fig. 1:**
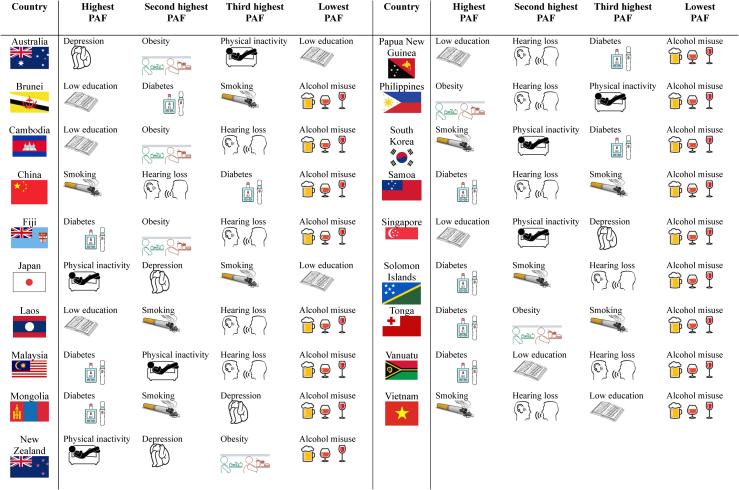
**Ranking of dementia risk factor weighted PAF values for each country in the WPR where data were available for at least seven risk factors. Figure shows the highest three and lowest PAF values**.

Because accounting for intercorrelation between risk factors is essential, the main results focus on weighted PAF estimates; unweighted values are presented in Appendix Table 5. Individual weighted PAFs varied widely across the WPR countries (Table 1). The greatest cross-country differences were observed for low education (range: 0.03–7.26; n = 16 countries) and obesity (range: 0.24–5.85; n = 28 countries). The smallest cross-country differences were observed for hypertension (range: 0.87–2.22; n = 27 countries) and alcohol misuse (range: 0.01–0.48; n = 24 countries). Fig. 1 shows the highest three and lowest PAF risk factor for each country where data was available for at least seven risk factors.

**Table 1:** Weighted individual PAF (%) values for each modifiable risk factor by country (n = 32).^d^

Blue indicates high-income, light orange indicates upper-middle and dark orange indicates lower-middle income countries.

^a^Highest PAF is the highest individual risk factor PAF value for the country shown.

^b^Lowest PAF is the lowest individual risk factor PAF value for the country shown.

^c^Range is the highest individual PAF minus the lowest individual PAF value for the country shown.

^d^Weighted PAFs were calculated using Lancet Commission communality estimates. For countries with fewer than nine available risk factors, these communality estimates were applied to the available risk factors to ensure consistency across countries.

Appendix Fig. 1A shows the 32 Western Pacific Region (WPR) countries included in the analysis. Appendix Fig. 1B–D presents heatmaps of weighted PAF values for all nine modifiable dementia risk factors. Substantial cross-country variation was observed, with clear differences in both the magnitude and distribution of risk factor burden across the region. Alcohol misuse showed uniformly very low weighted PAF values (<0.5%) across all countries. Marked heterogeneity was evident across the remaining risk factors. Low education and obesity demonstrated large between-country differences, with several lower-middle-income countries showing notably higher values than high-income settings. Depression, physical inactivity, and hypertension exhibited moderate PAF values with considerable variation across countries.

The heatmaps also illustrate distinct country-specific patterns. For example, smoking accounted for a disproportionately high burden in Kiribati and China, while physical inactivity had the greatest relative impact in the Marshall Islands and New Zealand. Depression and alcohol misuse contributed relatively more in Australia than in most other countries. While several risk factors contribute meaningfully to dementia risk across the region, their relative importance varies substantially between countries.

Thirteen countries had complete data for the seven-factor model: Australia, Brunei, Cambodia, Fiji, Japan, Malaysia, Mongolia, Papua New Guinea, Philippines, South Korea, Singapore, Vanuatu and Vietnam (Table 2). Unweighted combined PAFs ranged from 41.5% (Singapore) to 60.2% (Papua New Guinea). Weighted combined PAFs were substantially lower, ranging from 20 to 35%, with the highest values (>30%) in Papua New Guinea, Philippines, Vanuatu and Mongolia. These are consistent with published values (range: 24.5%–40.0%; pooled PAF (7–11) incorporating estimates from the USA, UK, Germany, Brazil, Portugal, Mozambique and globally) (Table 2).

**Table 2:** Unweighted and weighted combined PAF (%) values for the seven-factor model.

	Country/Region	Unweighted PAF, %	Weighted PAF, %
WPR countries	Australia	45.41	25.14
	Brunei	50.53	28.06
	Cambodia	43.78	22.60
	Fiji	47.51	25.92
	Japan	41.59	23.92
	Malaysia	44.31	24.29
	Mongolia	53.40	30.28
	Papua New Guinea	60.23	34.74
	Philippines	58.35	33.22
	South Korea	49.85	26.24
	Singapore	41.51	20.88
	Vanuatu	58.30	33.04
	Vietnam	42.12	21.93
Published estimates for non-WPR countries	Global	48.29	25.33
	USA	49.42	28.18
	Europe	52.75	30.59
	United Kingdom	53.85	31.31
	Germany	53.30	30.48
	Mozambique	43.98	24.45
	Brazil	55.30	32.29
	Portugal	65.72	40.09

### Income-stratified weighted PAFs

Table 3 shows weighted PAF values stratified by country income group. Clear differences were observed in both the magnitude and ranking of dementia risk factors across high-, upper-middle-, and lower-middle-income countries. Low education had the highest weighted PAF in lower-middle-income countries (mean 5.3%), significantly higher than in upper-middle- and high-income settings (ANOVA p = 0.0030). In contrast, diabetes ranked first in both high-income (4.7%) and upper-middle-income countries (4.9%) and remained high in lower-middle-income settings (4.7%).

**Table 3:** Mean weighted PAF (%) values, SDs, CIs and statistical analysis for each country income group (high, upper-middle and lower-middle) for each of the nine risk factors.

	Income group	N	Mean PAF (%)	SD	95% CI lower	95% CI upper	p-value	Post-hoc	p-value
Low education	High	7	1.29	1.79	−0.36	2.95	**0.0030**	H vs. L	**0.0242**^a^
	Upper-middle	5	1.34	1.00	0.10	2.58	N/A	H vs. U	0.4091
	Lower-middle	4	5.33	1.75	2.55	8.12	N/A	U vs. L	**0.0159**^a^
Obesity	High	11	3.25	2.14	1.82	4.69	0.4100	H vs. L	0.1509
	Upper-middle	10	2.32	1.71	1.09	3.54	N/A	H vs. U	0.4797
	Lower-middle	7	2.15	1.86	0.43	3.88	N/A	U vs. L	0.5362
Physical activity	High	10	2.55	0.90	1.90	3.19	0.1889	H vs. L	0.1331
	Upper-middle	10	2.08	0.89	1.45	2.72	N/A	H vs. U	0.2549
	Lower-middle	7	1.70	1.00	0.78	2.62	N/A	U vs. L	0.3023
Hypertension	High	12	1.49	0.32	1.29	1.70	**0.0066**	H vs. L	**0.0071**^a^
	Upper-middle	10	1.79	0.19	1.66	1.92	N/A	H vs. U	**0.0373**
	Lower-middle	5	1.90	0.12	1.75	2.04	N/A	U vs. L	0.0926
Diabetes	High	10	4.70	2.87	2.65	6.76	0.9806	H vs. L	0.9623
	Upper-middle	10	4.89	2.18	3.34	6.45	N/A	H vs. U	0.8383
	Lower-middle	7	4.71	1.77	3.07	6.34	N/A	U vs. L	0.7932
Smoking	High	9	2.16	0.57	1.72	2.60	**0.0035**	H vs. L	**0.0252**^a^
	Upper-middle	10	3.10	0.59	2.67	3.52	N/A	H vs. U	**0.0028**
	Lower-middle	6	3.29	0.80	2.46	4.13	N/A	U vs. L	0.4278
Depression	High	6	2.92	0.46	2.44	3.40	**0.0001**	H vs. L	**0.0012**^a^
	Upper-middle	8	2.31	0.26	2.10	2.53	N/A	H vs. U	**0.0153**^a^
	Lower-middle	7	1.97	0.10	1.89	2.06	N/A	U vs. L	**0.0037**^a^
Hearing loss	High	14	2.59	0.54	2.28	2.90	**0.0083**	H vs. L	**0.0000**^a^
	Upper-middle	10	3.19	0.46	2.86	3.52	N/A	H vs. U	**0.0001**^a^
	Lower-middle	7	3.46	0.87	2.65	4.27	N/A	U vs. L	**0.0338**^a^
Alcohol	High	8	0.31	0.19	0.15	0.47	0.2304	H vs. L	0.2415
	Upper-middle	9	0.20	0.15	0.09	0.31	N/A	H vs. U	0.1672
	Lower-middle	7	0.17	0.16	0.02	0.32	N/A	U vs. L	0.5360

a Bold value indicate statistically significant (i.e., <0.05) after Holm–Bonferroni correction for multiple pairwise comparisons within each risk factor.

Hearing loss showed significantly higher PAFs in upper-middle- and lower-middle-income countries (mean 3.2% and 3.5%) compared with high-income countries (p < 0.001), while smoking was substantially higher in upper-middle- and lower-middle-income groups (mean 3.1% and 3.3%) than in high-income countries (p = 0.0035). Depression showed the opposite pattern, with the highest PAFs in high-income countries (2.9%) and significantly lower values in upper-middle- and lower-middle-income settings (p = 0.0001).

PAFs for obesity, physical inactivity, hypertension, and alcohol misuse showed smaller between-group differences, although hypertension was significantly higher in lower-middle-income than in high-income countries (p = 0.0071). Following Holm–Bonferroni correction for multiple pairwise comparisons within each risk factor, differences between income groups remained statistically significant for low education (high vs lower-middle; upper-middle vs lower-middle), depression (all pairwise comparisons), hearing loss (all pairwise comparisons), and smoking (high vs upper-middle only). For hypertension, only the difference between high- and lower-middle-income groups remained significant after adjustment.

## Discussion

This study provides the most extensive assessment to date of modifiable dementia risk factors across the WPR, revealing substantial heterogeneity in both the magnitude and distribution of weighted PAFs. Across the WPR diabetes, hearing loss, and smoking showed the highest weighted PAF values on average, indicating that these risk factors contribute consistently to dementia burden across diverse country contexts. Although the combined seven-factor weighted PAF estimates across countries with complete data ranged from 20 to 35%, comparable to estimates from Europe, North America, South America, and Africa, the structure of risk across WPR countries differed markedly from global patterns.

Across the region, diabetes, hearing loss, and smoking consistently contributed most to dementia risk. Diabetes showed the highest weighted PAF in the high- and upper-middle income groups (4.7–4.9%), was also high in the lower-middle income group (4.7%), and exceeded 5% in several Pacific Island nations, reflecting poor metabolic health status affecting many WPR countries. The consistency of diabetes across income stratification suggests that the nutrition transition, rising obesity, and limited access to preventive services have affected the region.^15^^,^^16^ Hearing loss was the second most influential risk factor, with weighted PAFs ranging from 1.9% to 5.4%. Higher values were observed in upper-middle- and lower-middle-income countries, potentially reflecting reduced access to audiology services, screening, and hearing aid provision.^17^ Smoking ranked third region-wide, with PAFs ranging from 1.2% to 4.0%, showing large country-by-country differences that align with variation in tobacco control policies, commercial tobacco penetration, and enforcement capacity.^18^^,^^19^

Low education showed the most prominent income-related gradient. Weighted PAFs reached 7.3% in some lower-middle-income countries and averaged 5.3%, substantially higher than the 1.3% observed in upper-middle- and 1.3% in high-income settings. The substantially higher PAF for low education in lower-middle-income countries likely reflects persistent educational inequities across the region. The consistently high PAF for diabetes across income groups suggests that metabolic risk factors represent a region-wide driver of dementia risk in the Western Pacific. These disparities reflect historical and contemporary inequalities in schooling access, literacy, public investment in education, and gender equity in educational participation.^20^ Given that educational attainment is a foundational determinant of cognitive reserve,^21^ such inequalities have long-lasting implications for dementia burden and highlight a critical target for prevention.

Other risk factors showed more nuanced patterns. The higher depression-attributable PAF observed in high-income countries suggests a divergent mental-health burden across the region, potentially reflecting differences in diagnosis, reporting, and service access. Depression had the highest weighted PAFs in high-income settings (2.9%), consistent with greater diagnostic capacity, improved recognition, and higher health service utilisation in better-resourced systems. Hypertension showed a mild gradient from 1.5% in high-income to 1.9% in lower-middle-income countries, suggesting differences in screening, treatment adherence, and access to antihypertensive therapy.^22^ Obesity and physical inactivity displayed narrower income-related variation, but both remained important contributors to dementia risk, reflecting broader behavioural and lifestyle transitions occurring across the region. Alcohol misuse displayed uniformly low PAFs (<0.5%) across all income groups, a pattern distinct from global estimates and potentially shaped by cultural norms, regulatory environments, and the possibility of competing mortality in individuals with severe alcohol use disorders.^23^

In countries with sufficient data to model seven modifiable risk factors, weighted combined PAFs ranged from approximately 20% to 35%, substantially lower than unweighted estimates. The magnitude of weighted combined PAFs observed in the present study is broadly consistent with previously published estimates from high-income and global settings, which have reported pooled PAFs ranging from approximately 24.5% to 40.0% when modelling seven to eleven risk factors. Importantly, the region differed from global trends in several key areas. Diabetes PAFs were approximately double the global estimate (4.9% versus 2.3%) (6), reinforcing the urgency of coordinated regional action on metabolic risk. Low education and smoking displayed more extreme variability than observed globally (mean 4.5% and 2.3% respectively reported in the Lancet), highlighting the diversity of historical, cultural, and policy environments across the WPR.

The marked heterogeneity observed across countries underscores the need for dementia-prevention strategies tailored to national risk profiles, rather than reliance on region-wide or global averages. The implications for prevention strategy development are substantial. As only eight WPR countries have national dementia action plans, these findings offer a valuable evidence base for designing or refining national strategies. All countries should prioritise diabetes prevention and management, expansion of hearing care infrastructure, and stronger tobacco control. However, prevention efforts must diverge thereafter. Lower-middle-income countries should prioritise improvements in educational access and quality, alongside strengthened screening and management for hypertension and hearing loss. Upper-middle-income countries require robust tobacco-control measures, improved diabetes prevention, and expansion of auditory health services. High-income countries should integrate mental health assessment and care into primary and community health systems, while maintaining strong programmes for diabetes and hearing impairment.

Effective implementation will require overcoming structural, cultural, economic and logistical challenges. Many lower- and middle-income countries in the region face constraints in diagnostic capacity, health workforce availability, and specialist service provision. Innovative delivery models, such as task-shifting, mobile health diagnostics, community health worker-led programmes, and integration of dementia prevention within existing non-communicable disease frameworks, may offer feasible approaches. Addressing cultural perceptions of hearing impairment, mental health stigma, and barriers to help-seeking will also be essential for improving uptake of preventive services. Overall, these findings demonstrate that dementia risk factor profiles differ markedly by income level, emphasising that effective prevention will require approaches adapted to differing socioeconomic and health-system contexts.

This study has several strengths, including the most comprehensive application to date of PAF methodology across the WPR, the use of standardised relative risk and communality estimates, and the integration of country-specific prevalence data across nine modifiable risk factors. There are also limitations. Data completeness varied considerably, with only 13 countries having the full dataset for the seven-factor model and substantial missing data for low education and depression. Reliance on published rather than region-specific communality values may introduce misestimation, as risk factor intercorrelations could differ substantially across populations. Relative risk estimates were also assumed to be common across countries, although underlying risk relationships may vary due to contextual, demographic, and health-system factors. Temporal heterogeneity in prevalence datasets may also affect comparability, and exclusion of five Lancet Commission factors (high LDL cholesterol, traumatic brain injury, social isolation, air pollution, and untreated vision loss) likely leads to conservative estimates of total prevention potential.^24^ Direct comparison of prevalence-derived PAF estimates with previously published country-specific studies was not feasible due to heterogeneity in data sources, risk-factor definitions, and age cut-offs, underscoring the need for future analyses using harmonised national datasets. Further, PAF estimates based on binary exposure assume equivalence of risk across individuals and settings, which may obscure important variation related to duration of exposure, age at onset, treatment adequacy, and health system access. These limitations are particularly relevant when comparing countries with markedly different care pathways and preventive infrastructure. An additional limitation relates to the use of communality estimates derived from non-regional data. Risk factor clustering is known to vary substantially by socioeconomic context, culture, and health system characteristics,^25^ and applying a single communality structure across diverse Western Pacific populations may lead to over- or under-estimation of combined PAFs in some settings. This variability in risk factor overlap has been demonstrated in prior work, including Mukadam and colleagues (2019), who reported substantial differences in nine-factor clustering across China, India, and Latin American settings.

Future research should focus on improving data completeness, generating region- and country-specific communality estimates, evaluating the cost-effectiveness of prevention strategies in diverse settings, and testing culturally adapted interventions for hearing loss, depression, smoking cessation, and diabetes control. Longitudinal implementation research will be essential to determine the real-world effectiveness of dementia-prevention programmes in settings with varying health-system capacity. Access to harmonised, age-specific prevalence data across countries and risk factors will allow more refined age-stratified PAF estimates and improve comparability across the WPR.

This study provides a robust epidemiological foundation for dementia-prevention planning across the WPR. While diabetes, hearing loss, and smoking represent universal regional priorities, large cross-country differences, particularly in low education, depression, obesity, and physical inactivity, highlight the need for locally tailored national strategies aligned with each country's epidemiological profile, cultural context, and resource base. The observed 20–35% combined prevention potential underscores the considerable opportunity to mitigate future dementia burden. Realising this potential will depend on moving beyond global one-size-fits-all recommendations toward bespoke, context-specific prevention strategies capable of addressing the needs of one of the world's most diverse and rapidly ageing regions.

## Contributors

CB and BS designed the study. CB and BS directly accessed and verified the underlying data. CB drafted the manuscript. HS, JD, SN, KA, TB, MS, and BS provided critical review of the content and editing. MA produced the heatmap figures. All authors reviewed and edited the manuscript and approved the final version. CB and BS were responsible for the decision to submit the manuscript.

## Data sharing statement

This study is based primarily on publicly available, aggregated secondary data. Any additional data generated during the study are available from the corresponding author upon reasonable request.

## Editor note

*The Lancet Group* takes a neutral position with respect to territorial claims in published maps and institutional affiliations.

## Declaration of interests

Kaarin J. Anstey has grants or contracts with the NHMRC, Australian Research Council, and the Wellcome Trust. Kaarin J. Anstey also received an honorarium from Eli Lilley. Tanya Buchanan is CEO of Dementia Australia. All remaining authors declare no competing interests.

## References

[1] Clarke A.J., Brodtmann A., Irish M. (2024). Risk factors for the neurodegenerative dementias in the Western Pacific region. Lancet Reg Health West Pac.

[2] World Health Organization Existence of national dementia plans (GHO indicator). https://www.who.int/data/gho/data/indicators/indicator-details/GHO/existence-of-dementia-national-plan.

[3] Timmins H.C., Mok V.C., Kim S.H. (2024). Regional health priorities for dementia: a roadmap for the Western Pacific. Lancet Reg Health West Pac.

[4] Alzheimer's Disease International World Alzheimer report 2024: global changes in attitudes to dementia. https://www.alzint.org/resource/world-alzheimer-report-2024/.

[5] Stephan B.C.M., Cochrane L., Kafadar A.H. (2024). Population attributable fractions of modifiable risk factors for dementia: a systematic review and meta-analysis. Lancet Healthy Longev.

[6] Livingston G., Huntley J., Liu K.Y. (2024). Dementia prevention, intervention, and care: 2024 report of the Lancet standing Commission. Lancet.

[7] Ashby-Mitchell K., Burns R., Shaw J., Anstey K.J. (2017). Proportion of dementia in Australia explained by common modifiable risk factors. Alzheimers Res Ther.

[8] Luck T., Riedel-Heller S.G. (2016). Prävention von Alzheimer-Demenz in Deutschland. Nervenarzt.

[9] Mayer F., Di Pucchio A., Lacorte E. (2018). Attributable cases of Alzheimer disease and vascular dementia due to modifiable risk factors in Europe and Italy. Dement Geriatr Cogn Dis Extra.

[10] Norton S., Matthews F.E., Barnes D.E., Yaffe K., Brayne C. (2014). Potential for primary prevention of Alzheimer's disease: analysis of population-based data. Lancet Neurol.

[11] Oliveira D., Otuyama L.J., Mabunda D. (2019). Reducing the number of people with dementia through primary prevention in Mozambique, Brazil, and Portugal. J Alzheimers Dis.

[12] Levin M.L. (1953). The occurrence of lung cancer in man. Acta Unio Int Contra Cancrum.

[13] World Bank World Bank country and lending groups. https://datahelpdesk.worldbank.org/knowledgebase/articles/906519-world-bank-country-and-lending-groups.

[14] StataCorp (2023).

[15] Peng W., Zhang L., Wen F. (2024). Trends and disparities in non-communicable diseases in the Western Pacific region. Lancet Reg Health West Pac.

[16] Tong T.J., Mohammadnezhad M., Alqahtani N.S. (2022). Determinants of overweight and obesity and preventive strategies in Pacific countries: a systematic review. Glob Health J.

[17] Waterworth C.J., Marella M., O'Donovan J., Bright T., Dowell R., Bhutta M.F. (2022). Barriers to access to ear and hearing care services in low- and middle-income countries: a scoping review. Glob Public Health.

[18] Mackay J.M., Dorotheo E.U., Assunta M., Ritthiphakdee B. (2022). Tobacco control in Asia-Pacific: wins, challenges and targets. Tob Control.

[19] Odo D.B., Semos I., Toloube O. (2025). Framework convention on tobacco control progress in Pacific Island countries (2007–2023): a comprehensive review. Tob Control.

[20] Smith W.C., Voigt A., Zhang Y. (2021). Barriers to Secondary Education in the Asia-Pacific Region: a Scoping Review of Four Countries. Final Report of the Scotland Funding Council GCRF Project Universal Secondary Education in the Asia Pacific Region.

[21] Kim Y., Stern Y., Seo S.W. (2024). Factors associated with cognitive reserve according to education level. Alzheimers Dement.

[22] Bruyn E., Nguyen L., Schutte A.E., Murphy A., Perel P., Webster R. (2022). Implementing single-pill combination therapy for hypertension: health system requirements in 30 LMICs. Glob Heart.

[23] Ding C., O'Neill D., Bell S., Stamatakis E., Britton A. (2021). Association of alcohol consumption with morbidity and mortality in cardiovascular disease: original data and meta-analysis. BMC Med.

[24] Rolandi E., Zaccaria D., Vaccaro R. (2020). Estimating dementia prevention potential through elimination of modifiable risk factors: a population-based study. Alzheimers Res Ther.

[25] Mukadam N., Sommerlad A., Huntley J., Livingston G. (2019). Population attributable fractions for risk factors for dementia in low-income and middle-income countries: an analysis using cross-sectional survey data. Lancet Glob Health.

