# Supplementary material for: Country-specific modifiable dementia risk factors across the Western Pacific Region determined by population attributable fraction
Source: Lancet Reg Health West Pac. 2026 Apr 17;69:101857. doi: 10.1016/j.lanwpc.2026.101857 (PMC13098443; doi:10.1016/j.lanwpc.2026.101857)
Supplement: Appendix Tables and Figures [file mmc1.docx]

**Appendix Material**

**Title** Country-specific modifiable dementia risk factors across the Western Pacific Region determined by population attributable fraction

**Authors** *Claire V Burley^1,2^, Hamid R. Sohrabi^3,4^, Maha SM Alshahrani^1,^ Jennifer Dunne^1^, Sharon L Naismith^5^, Kaarin J Anstey^6,7,8^, Tanya Buchanan^9^, Mario Siervo^1,10^, & Blossom CM Stephan^1^.

*^1^Dementia Centre of Excellence, enAble Institute, Curtin University, Perth, Western Australia, Australia*

*^2^School of Health Sciences, University of New South Wales, Kensington, New South Wales, Australia*

*^3^Centre for Healthy Ageing, Health Futures Institute, Murdoch University, Perth, Western Australia, Australia*

*^4^School of Psychology, Murdoch University, Perth, Western Australia, Australia*

^5^*Brain and Mind Centre, The University of Sydney, Sydney, New South Wales, Australia*

*^6^School of Psychology, University of New South Wales, Kensington, New South Wales, Australia*

*^7^Neuroscience Research Australia, 139 Barker St, Randwick, 2031, Australia*

*^8^University of New South Wales, Ageing Futures Institute, Kensington, New South Wales, Australia*

*^9^Dementia Australia, North Ryde, New South Wales, Australia*

*^10^School of Population Health, Curtin University, Perth, Australia*

**Appendix Table 1. Risk factor prevalence data sources.**

| **Risk Factor(s)** | **Data Source** | **Year/Period** | **Link/Reference** |
| --- | --- | --- | --- |
| Obesity, physical inactivity, hypertension, diabetes, and alcohol use | World Health Organization, Western Pacific Health Data Platform | Various | https://data.wpro.who.int |
| Smoking | Web Annex VI: Global Tobacco Control Policy Data | 2021 | <https://www.who.int/publications/i/item/WHO-HEP-HPR-TFI-2021.10> |
| Depression | Mental Health Atlas WHO Global Health Estimates | Various | <https://www.who.int/data/gho/indicator-metadata-registry/imr-details/5281> |
| Low education | Robert J. Barro and Jong-Wha Lee | Various | http://www.barrolee.com/; Education Statistics (<https://data.worldbank.org/> |
| Hearing loss | Supplement to: GBD 2019 Hearing Loss Collaborators. Hearing loss prevalence and years lived with disability, 1990–2019: findings from the Global Burden of Disease Study 2019. Lancet 2021; 397: 996–1009. Limited education = people aged 15 years+ with no formal education 2010. | 1990-2019; Education 2010 | [https://pubmed.ncbi.nlm.nih.gov/3 3714390/](https://pubmed.ncbi.nlm.nih.gov/3%203714390/) |

**Appendix Table 2. Definitions and cut-offs for modifiable dementia risk factors.**

| **Risk factor** | **Definition** | **Cut-off** |
| --- | --- | --- |
| Low education | Limited formal educational attainment | No formal education (≥15 years) |
| Obesity | Excess body weight | Body mass index (BMI) ≥30 kg/m² |
| Physical inactivity | Insufficient physical activity for health | <150 minutes/week of moderate-intensity physical activity (or equivalent) |
| Hypertension | Elevated blood pressure | Systolic blood pressure ≥140 mmHg and/or diastolic blood pressure ≥90 mmHg |
| Diabetes | Diagnosed diabetes mellitus | Self-reported or clinically diagnosed diabetes (type not distinguished) |
| Smoking | Tobacco smoking | Current tobacco smoking (daily or non-daily) |
| Depression | Depressive disorders | Presence of depressive disorder based on population-level estimates |
| Hearing loss | Reduced hearing acuity | ≥20 dB hearing loss in the better-hearing ear |
| Excessive alcohol use | Harmful or hazardous alcohol consumption | >21 UK units/week (or equivalent) |

**Appendix Table 3. Risk factor prevalence (%) data for nine established dementia risk factors for 32 countries within the Western Pacific Region (WPR). Table shows pattern of missing data.**

|  |  |  |  |  |  |  |  |  |  |  |
| --- | --- | --- | --- | --- | --- | --- | --- | --- | --- | --- |
| **Prevalence (%)** |  | **Dementia risk factors and relative risk (RR)** | | | | | | | | |
| **Country** |  | **Low Education** | **Obesity** | **Inactivity** | **Hypertension** | **Diabetes** | **Smoking** | **Hearing loss** | **Depression** | **Alcohol Misuse** |
|  |  | **1.6** | **1.3** | **1.2** | **1.2** | **1.7** | **1.3** | **1.4** | **2.2** | **1.2** |
| American Samoa | H | - | - | - | - | - | - | 21.2 | - | - |
| Australia | H | 0.7 | 29.0 | 29.0 | 15.2 | 5.9 | 13.1 | 13.5 | 5.9 | 10.5 |
| Brunei | H | 21.1 | 14.1 | 27.3 | 18.9 | 9.4 | 22.8 | 13.5 | 4.0 | 0.2 |
| Cambodia | LM | 22.2 | 3.9 | 10.5 | 26.1 | 7.1 | 26.5 | 22.0 | 3.4 | 6.6 |
| China | UM | - | 6.2 | 14.1 | 19.2 | 8.8 | 41.1 | 22.6 | 4.2 | 7.1 |
| Cook Islands | H | - | 55.9 | 18.5 | 22.3 | 27.5 | 22.7 | 21.0 | - | - |
| Fiji | UM | 2.4 | 30.2 | 17.4 | 21.7 | 17.4 | 19.1 | 21.3 | 3.5 | 3.3 |
| French Polynesia | H | - | - | - | 17.6 | - | - | - | - | - |
| Guam | H | - | - | - | 21.5 | - | - | 21.0 | - | - |
| Japan | H | 0.1 | 4.3 | 35.5 | 24.8 | 6.7 | 26.3 | 13.6 | 4.2 | 8.0 |
| Kiribati | LM | - | 46.0 | 40.4 | - | 22.3 | 43.0 | 21.7 | 3.1 | 0.5 |
| Laos | LM | 31.2 | 5.3 | 16.3 | - | 7.7 | 38.4 | 22.0 | 3.2 | 10.7 |
| Malaysia | UM | 6.9 | 15.6 | 38.8 | 22.9 | 11.7 | 32.1 | 21.5 | 3.8 | 0.9 |
| Marshall Islands | UM | - | 52.9 | 43.5 | 21.3 | 21.1 | 32.3 | 21.5 | - | - |
| Micronesia | LM | - | 45.8 | 36.6 | 25.0 | 22.0 | - | 21.5 | 3.1 | 2.5 |
| Mongolia | UM | 4.3 | 20.6 | 18.6 | 29.0 | 11.7 | 40.3 | 18.0 | 4.2 | 8.2 |
| Nauru | H | - | 61.0 | 42.1 | 20.5 | 29.2 | 29.5 | 21.3 | - | 3.7 |
| New Zealand | H | - | 30.8 | 42.4 | 16.2 | 6.9 | 12.8 | 13.0 | 5.4 | 10.6 |
| Niue | UM | 0.9 | 50.0 | 6.9 | 24.2 | 27.1 | - | 21.2 | - | 10.7 |
| Northern Mariana Islands | H | - | - | - | - | - | - | 21.1 | - | - |
| Palau | H | - | 55.3 | 40.9 | 22.9 | 23.2 | 20.5 | 20.5 | - | - |
| Papua New Guinea | LM | 37.9 | 21.3 | 14.8 | 25.6 | 14.8 | 40.4 | 40.4 | 3.0 | 1.4 |
| Philippines | LM | 2.7 | 6.4 | 39.7 | 22.6 | 7.2 | 31.8 | 31.8 | 3.3 | 6.9 |
| Republic of Korea | H | 3.4 | 4.7 | 35.4 | 11.0 | 8.0 | 29.8 | 13.4 | 4.1 | 9.7 |
| Samoa | LM | - | 6.1 | 12.6 | 24.0 | 24.6 | 25.0 | 21.5 | 3.2 | 2.7 |
| Singapore | H | 15.3 | 6.1 | 36.5 | 14.6 | 7.9 | 20.5 | 13.5 | 4.6 | 2.0 |
| Solomon Islands | LM | - | 22.5 | 18.2 | 22.0 | 13.9 | 40.0 | 21.8 | 2.9 | 1.8 |
| Tokelau | LM | - | - | - | - | - | - | 21.3 | - | - |
| Tonga | UM | 0.7 | 48.2 | 17.4 | 23.7 | 24.2 | 37.4 | 21.3 | 3.2 | 0.8 |
| Tuvalu | UM | - | 51.6 | 27.3 | 23.7 | 23.8 | 30.2 | 21.4 | - | 1.5 |
| Vanuatu | LM | - | 25.2 | 8 | 24.2 | 15.9 | 20.5 | 21.6 | 3.1 | 2.3 |
| Vietnam | LM | 15.2 | 2.1 | 25.4 | 23.4 | 5.3 | 35.2 | 21.4 | 4.0 | 8.7 |
|  |  |  |  |  |  |  |  |  |  |  |
| Global (Livingston^2^) |  | 23.2 | 13.0 | 27.5 | 31.1 | 9.3 | 22.3 | 59.0 | 7.2 | 13.3 |
|  |  |  |  |  |  |  |  |  |  |  |

**Abbreviations** H=High income countries, LM=Lower-middle income countries, UM=Upper-middle income countries.

**Key** ‘-’=Insufficient data for PAF calculation.

**Appendix Table 4. Relative risk and communality values for each dementia risk factor from the 2024 Lancet Commission and Norton 7-Factor Model.**

|  | **Lancet** |  | **Norton** |  |
| --- | --- | --- | --- | --- |
| **Risk Factor** | **Relative Risk** | **Communality %** | **Relative Risk** | **Communality %** |
| **Low education** | 1.60 | 0.608 | 1.59 | 0.456 |
| **Obesity** | 1.30 | 0.622 | 1.60 | 0.437 |
| **Inactivity** | 1.20 | 0.567 | 1.82 | 0.490 |
| **Hypertension** | 1.20 | 0.595 | 1.61 | 0.650 |
| **Diabetes** | 1.70 | 0.493 | 1.46 | 0.509 |
| **Smoking** | 1.30 | 0.650 | 1.59 | 0.581 |
| **Depression** | 2.20 | 0.452 | 1.65 | 0.374 |
| **Hearing loss** | 1.40 | 0.609 |  |  |
| **Alcohol** | 1.20 | 0.772 |  |  |

**Appendix Table 5. Population attributable fraction population attributable risk (PAF) data for nine established dementia risk factors for 32 countries within the Western Pacific Region (WPR) countries. Table shows pattern of missing data.**

| **PAF values (unweighted)** | |  | **Dementia risk factors and relative risk (RR)** | | | | | | | | |
| --- | --- | --- | --- | --- | --- | --- | --- | --- | --- | --- | --- |
| **Country** | **Income Level** | | **Low education** | **Obesity** | **Inactivity** | **Hypertension** | **Diabetes** | **Smoking** | **Hearing loss** | **Depression** | **Alcohol Misuse** |
|  |  | | **1.6** | **1.3** | **1.2** | **1.2** | **1.7** | **1.3** | **1.4** | **2.2** | **1.2** |
| American Samoa | | H |  | - | - | - | - | - | 7.82 | - | - |
| Australia | | H | 0.42 | 8.00 | 5.48 | 2.95 | 3.97 | 3.78 | 5.12 | 6.61 | 2.06 |
| Brunei | | H | 11.24 | 4.06 | 5.18 | 3.64 | 6.17 | 6.40 | 5.12 | 4.58 | 0.04 |
| Cambodia | | LM | 11.75 | 1.16 | 2.06 | 4.96 | 4.73 | 7.36 | 8.09 | 3.92 | 1.30 |
| China | | UM | - | 1.83 | 2.74 | 3.70 | 5.80 | 10.98 | 8.29 | 4.80 | 1.40 |
| Cook Islands | | H | - | 14.36 | 3.57 | 4.27 | 16.14 | 6.38 | 7.75 | - | - |
| Fiji | | UM | 1.42 | 8.31 | 3.36 | 4.16 | 10.86 | 5.42 | 7.85 | 4.03 | 0.66 |
| French Polynesia | | H | - | - | - | 3.40 | - | - | - | - | - |
| Guam | | H | - | - | - | 4.12 | - | - | - | - | - |
| Japan | | H | 0.08 | 1.27 | 6.63 | 4.73 | 4.48 | 7.31 | 5.16 | 4.80 | 1.57 |
| Kiribati | | LM | - | 12.13 | 7.48 | - | 13.50 | 11.43 | 7.99 | 3.59 | 0.10 |
| Laos | | LM | 15.77 | 1.57 | 3.16 | - | 5.11 | 10.33 | 8.09 | 3.70 | 2.10 |
| Malaysia | | UM | 3.98 | 4.47 | 7.20 | 4.38 | 7.57 | 8.78 | 7.92 | 4.36 | 0.18 |
| Marshall Islands | | UM | - | 13.70 | 8.00 | 4.09 | 12.87 | 8.83 | 7.92 | - | - |
| Micronesia | | LM | - | 12.08 | 6.82 | 4.76 | 13.34 | - | 7.92 | 3.59 | 0.50 |
| Mongolia | | UM | 2.52 | 5.82 | 3.59 | 5.48 | 7.57 | 10.79 | 6.72 | 4.80 | 1.61 |
| Nauru | | H | - | 15.47 | 7.77 | 3.94 | 16.97 | 8.13 | 7.85 | - | 0.73 |
| New Zealand | | H | - | 8.46 | 7.82 | 3.14 | 4.61 | 3.70 | 4.94 | 6.09 | 2.08 |
| Niue | | UM | 0.54 | 13.04 | 1.36 | 4.62 | 15.95 | - | 7.82 | - | 2.10 |
| Northern Mariana Islands | | H | - | - | - | - | - | - | 7.78 | - | - |
| Palau | | H | - | 14.23 | 7.56 | 4.38 | 13.97 | 5.79 | 7.58 | - | - |
| Papua New Guinea | | LM | 18.53 | 6.01 | 2.87 | 4.87 | 9.39 | 10.81 | 13.91 | 3.47 | 0.28 |
| Philippines | | LM | 1.59 | 1.88 | 7.36 | 4.32 | 4.80 | 8.71 | 11.28 | 3.81 | 1.36 |
| Republic of Korea | | H | 2.00 | 1.39 | 6.61 | 2.15 | 5.30 | 8.21 | 5.09 | 4.69 | 1.90 |
| Samoa | | LM | - | 1.80 | 2.46 | 4.58 | 14.69 | 6.98 | 7.92 | 3.70 | 0.54 |
| Singapore | | H | 8.41 | 1.80 | 6.80 | 2.84 | 5.24 | 5.79 | 5.12 | 5.23 | 0.40 |
| Solomon Islands | | LM | - | 6.32 | 3.51 | 4.21 | 8.87 | 10.71 | 8.02 | 3.36 | 0.36 |
| Tokelau | | LM | - | - | - | - | - | - | 7.85 | - | - |
| Tonga | | UM | 0.42 | 12.63 | 3.36 | 4.53 | 14.49 | 10.09 | 7.85 | 3.70 | 0.16 |
| Tuvalu | | UM | - | 13.40 | 5.18 | 4.53 | 14.28 | 8.31 | 7.89 | - | 0.30 |
| Vanuatu | | LM | - | 7.03 | 1.57 | 4.62 | 10.02 | 5.79 | 7.95 | 3.59 | 0.46 |
| Vietnam | | LM | 8.36 | 0.63 | 4.83 | 4.47 | 3.58 | 9.55 | 7.89 | 4.58 | 1.71 |
|  | |  |  |  |  |  |  |  |  |  |  |
| Global (Livingston) | |  | 12.22 | 3.75 | 5.21 | 5.86 | 6.11 | 6.27 | 19.09 | 7.95 | 2.59 |
|  | |  |  |  |  |  |  |  |  |  |  |

**Abbreviations** H=High income countries, LM=Lower-middle income countries, UM=Upper-middle income countries. ‘-’=Insufficient data for PAF calculation.

**Appendix Figure 1A. Map of countries included in the WHO Western Pacific Region. *Map shown for geographical reference only.***


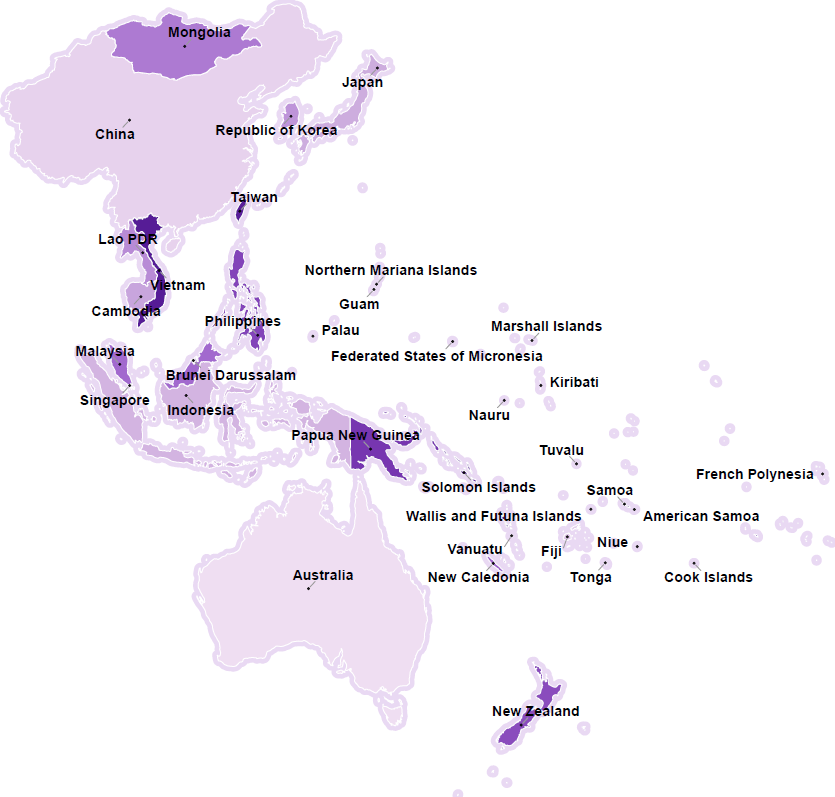


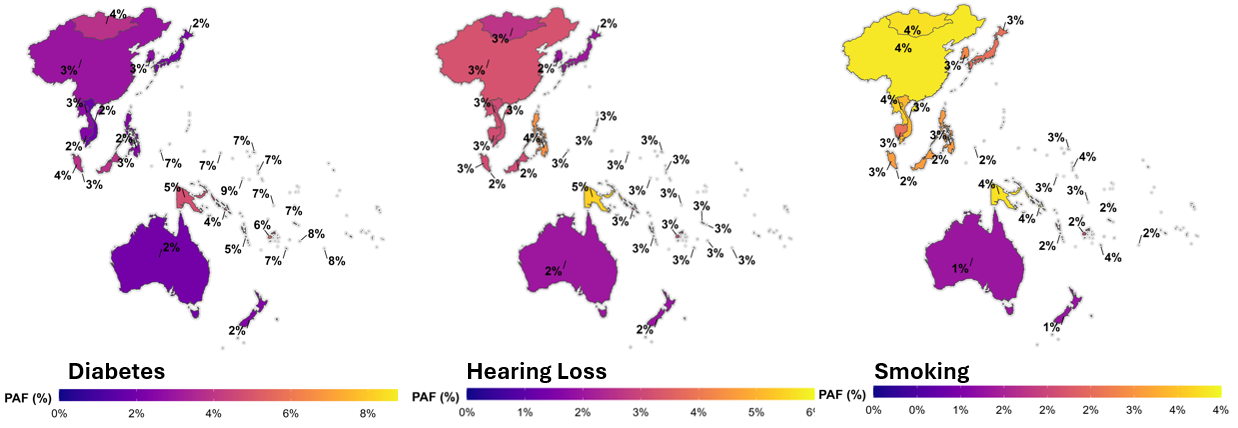


**Appendix Figure 1B. Heatmaps showing weighted population attributable fractions (PAFs) for diabetes, hearing loss and smoking across Western Pacific Region countries.**


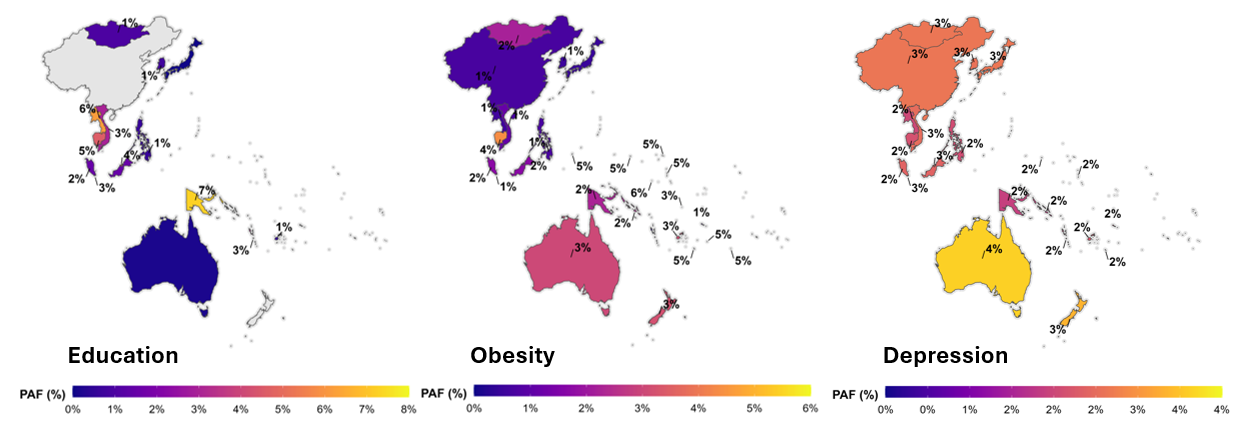


**Appendix Figure 1C. Heatmaps showing weighted population attributable fractions (PAFs) for low education, obesity and depression across Western Pacific Region countries.**


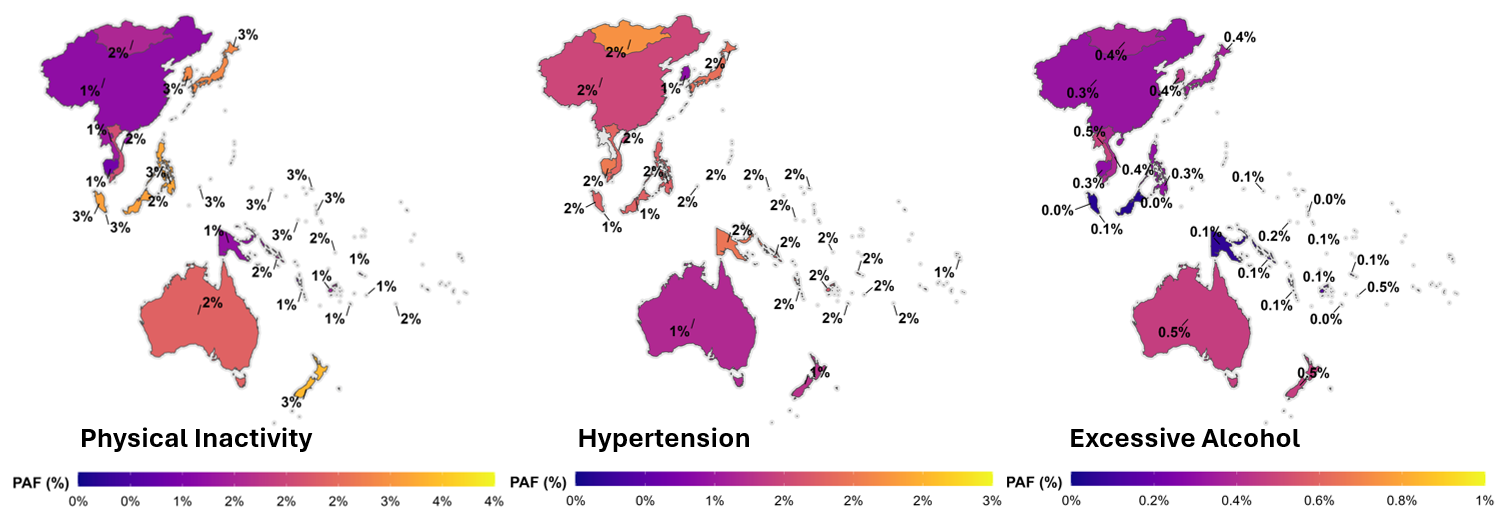


**Appendix Figure 1D. Heatmaps showing weighted population attributable fractions (PAFs) for physical inactivity, hypertension and excessive alcohol across Western Pacific Region countries.**

Footnote. Maps are ordered by risk factor severity (left to right): diabetes, hearing loss, smoking, low education, obesity, depression, physical inactivity, hypertension, and excessive alcohol. Darker colour corresponds with higher PAF values, indicating countries that would benefit most by targeting that risk factor. Colour scales are standardized within each risk factor to show relative country differences.
